# The fate of water within Earth and super-Earths and implications for plate tectonics

**DOI:** 10.1098/rsta.2015.0394

**Published:** 2017-04-17

**Authors:** Sonia M. Tikoo, Linda T. Elkins-Tanton

**Affiliations:** 1 Department of Earth and Planetary Sciences, Rutgers University, 610 Taylor Road, Piscataway, NJ 08854, USA; 2 School of Earth and Space Exploration, Arizona State University, 781 Terrace Mall, Tempe, AZ 84287, USA

**Keywords:** water, planet formation, plate tectonics, Magma Ocean, extrasolar planets, super-Earths

## Abstract

The Earth is likely to have acquired most of its water during accretion. Internal heat of planetesimals by short-lived radioisotopes would have caused some water loss, but impacts into planetesimals were insufficiently energetic to produce further drying. Water is thought to be critical for the development of plate tectonics, because it lowers viscosities in the asthenosphere, enabling subduction. The following issue persists: if water is necessary for plate tectonics, but subduction itself hydrates the upper mantle, how is the upper mantle initially hydrated? The giant impacts of late accretion created magma lakes and oceans, which degassed during solidification to produce a heavy atmosphere. However, some water would have remained in the mantle, trapped within crystallographic defects in nominally anhydrous minerals. In this paper, we present models demonstrating that processes associated with magma ocean solidification and overturn may segregate sufficient quantities of water within the upper mantle to induce partial melting and produce a damp asthenosphere, thereby facilitating plate tectonics and, in turn, the habitability of Earth-like extrasolar planets.

This article is part of the themed issue ‘The origin, history and role of water in the evolution of the inner Solar System’.

## 1. Introduction

At present, the small fraction of water in Earth's asthenosphere is replenished by subduction. However, how the upper mantle was initially hydrated remains unresolved. The primordial Earth is thought to have accumulated water through two possible avenues: original accretion of hydrous material or delivery through a late accretion following the Moon-forming impact. While the presence of metallic cores in terrestrial planets suggests that early accretion may have taken place in a reducing environment with very little water [1], meteorite compositions as well as the certainty of radial mixing during accretion suggest that the terrestrial planets accreted with some non-zero water content [2,3]. The transition from planetesimal accretion to oligarchic growth led to the expansion of the proto-Earth's accretion feeding zone to distances spanning the outer asteroid belt and perhaps even the orbits of Jupiter and Saturn [4,5]. A majority of terrestrial water may have been carried by a few planetary embryos from the outer asteroid belt, which then combined to form the Earth during the final stages of planetary accretion [6].

Giant impact events associated with the final phase of planetary formation may lead to the creation of one or more partial silicate mantle magma oceans on terrestrial planets [7–10]. The heat released during accretion and core formation is sufficient to melt the Earth [11,12]. An impact of a similar scale as the Moon-forming event [13] may produce a whole-mantle magma ocean. Although whole-mantle magma oceans are thus far unproven via geochemical evidence, constraints from various incompatible elements suggest that the early Earth may have experienced fractional crystallization of at least one magma ocean. Tucker & Mukhopadhyay [14] found that fractional crystallization and outgassing of at least two giant impact-induced magma oceans are required to explain the elevated ^3^He/^22^Ne ratios of mid-ocean ridge basalts relative to that of the solar nebula. Fractional crystallization of a magma ocean may also be responsible for heterogeneities in ^142^Nd and ^182^W [15] and He and Ne isotopes [16] in mantle source regions.

Magma ocean processes are detailed in Abe [17,18], Solomatov [11], Elkins-Tanton *et al*. [19] and Elkins-Tanton [20,21]. For Earth-sized planets, magma oceans may solidify from the bottom-up because the solidus and adiabat intersect at depth [11,12,20]. Alternatively, magma oceans may solidify both upward and downward from the mid-mantle due to density inversions between solidifying phases and residual liquid where liquids sink, creating basal magma oceans (summarized in Elkins-Tanton [21]). Fractional solidification of a magma ocean produces compositional heterogeneity in the resulting solid mantle; it is a silicate differentiation event for the planet.

Density stratification is also produced during fractional solidification. Because the magnesium ion is smaller than that of iron, magnesium is preferentially incorporated into mantle silicate minerals during crystallization. The remaining magma ocean liquid becomes increasingly rich in dense iron and other incompatible elements, yielding an unstable mantle profile with density increasing towards the planet exterior as solidification progresses from the bottom up. As a result, the solid mantle overturns until it reaches a gravitationally stable configuration. Even shallow fractionated magma oceans will overturn if there is underlying undifferentiated material warm enough to allow for sinking and reorganization, because the density of the final solids will be higher than the average bulk density. Finally, the planet conducts heat through the mantle and radiates it through the newly formed atmosphere and ultimately into space.

In the simplest case, the overturned solid mantle is compositionally stable and resistant to thermal convection. During this early period, before the onset of thermal convection, a solid conductive lid would grow near the planetary surface and hinder the onset of plate tectonics. Time is required for this cold boundary to overcome compositional stability at the top of the mantle and initiate small-scale thermal convection there. Thickening of a conductive lid at the planetary surface would grow at a rate proportional to (*κt*)^1/2^, where *κ* represents the diffusivity of the conductive lid and *t* represents time. The conductive lid will continue to grow until it reaches its critical Rayleigh number and becomes unstable, at which time the onset of convection takes place (estimated to be approx. 25–100 Myr after magma ocean solidification and overturn [22]). Similarly, the bottom boundary of the mantle would require significant heating from the core before thermal buoyancy would overcome compositional stability there.

In this paper, we first describe how water can be delivered to a growing Earth and retained through the high-energy processes of melting and differentiation. We then track water through the magma ocean solidification process, as the majority of water is expelled onto the planetary surface, while a small but critical fraction is retained in the now-solid mantle. This newly solidified mantle will overturn through compositional density instability. We subsequently describe a process by which magma ocean overturn may have caused sufficient enrichment of water in the upper mantle of the primordial Earth to facilitate the onset of convection and, in turn, plate tectonic activity. This process should also occur for greater than or equal to 1 Earth mass (*M*_E_) terrestrial extrasolar planets. Plate tectonic activity on extrasolar planets would play an integral role in regulating surface and atmospheric conditions and would therefore support long-term planetary habitability.

## 2. Evidence for water retention during primary planetary formation

Water, or hydroxyl (OH), was delivered by planetesimals and planetary embryos to the growing Earth during primary accretion (here defined as the period the Earth obtained most of its mass, ending with the Moon-forming impact). Despite the high temperatures that may have been achieved in planetesimals by radiogenic decay of ^26^Al [23,24], models do not show a complete loss of water from planetesimals [25–27]. Even meteorites from differentiated bodies show traces of retained water [2], though analyses of nominally anhydrous minerals in these meteorites should be done for confirmation. Additionally, very little water is needed to make a wet planetary surface. If homogeneously mixed into the mantle, the Earth's hydrosphere adds only about 230 ppm (0.023 wt%) of water. With as little as 500 ppm of water in the Earth's magma ocean, the solidified and cooled planet would have a damp mantle and sufficient surface water for the habitable planet we have today.

Arguing that the Earth (and other terrestrial planets) was accreted from materials with a trace of water is not sufficient, however. The planets need to have been able to retain traces of water in their interiors and on their surfaces through the processes of magma ocean solidification and steam atmosphere cooling and collapse. Here, a combination of sample analyses and modelling helps support the hypothesis of water retention through the giant impact stage of accretion.

The Moon was long thought to be almost completely dry, or at least, to possess in bulk several orders of magnitude less water than does the Earth. The discovery of water in volcanic glass beads shows conclusively that the melting source regions of the magmas contain water [28,29]. The bulk Moon is not likely to contain more than about 100 ppm water [28–30], but this water is heterogeneously distributed (as would be expected from magma ocean solidification), leaving some melting source regions damp. The source regions of the lunar picritic glasses vary from about 200 to about 500 km depth [31]. Recent modelling of the physical conditions of the Moon-forming process further support the retention of even the most volatile species at some level [32,33]. Thus, the Moon-forming impact did not fully dry the Moon.

Following the solidification of the magma ocean and the production of a steam atmosphere, the planet cools and the atmosphere collapses into surface water [17,18,20,34,35]. Water can be stripped from the atmosphere by an active Sun before collapse from steam to water [36], but that did not likely happen to the Earth because of its gravitational field and distance from the Sun. Furthermore, models show that atmospheric stripping by the tail of accretion is inefficient [37,38].

We therefore argue that terrestrial planets are accreted with sufficient water to produce a damp mantle and a wet surface. Though planets inevitably sustain a tail of accretion that delivers additional water and other elements and molecules, this tail of accretion is not necessary to produce a habitable planet. The models presented hereafter proceed from a newly solidified Earth with a damp mantle.

## 3. Magma ocean models

### (a) Model framework

A variety of geochemical studies suggest that the Earth is likely to have accreted from planetesimals of chondritic compositions (see review by Drake & Righter [39]). In particular, enstatite chondrites provide the closest oxygen isotopic match to the bulk Earth [40]. Chondritic meteorites have variably been found to contain up to 20 wt% water [3], while certain achondrites may contain as much as approximately 3 wt% water [2], although most meteorites are drier. Because impact models show that melting from planetesimal collisions occurs internally preferentially to externally [41,42], it is likely that volatiles were at least partially retained through the final stages of planet formation. In view of this, we model scenarios for terrestrial magma ocean solidification and overturn using initial water contents spanning from 0.001 wt%–5 wt%. We model magma ocean depths ranging between 250 and 2800 km (i.e. nearly whole mantle). We employ the same code previously used for modelling a magma ocean for the primordial Earth in Elkins-Tanton [20] and Brown *et al*. [43].

**Figure 1.**
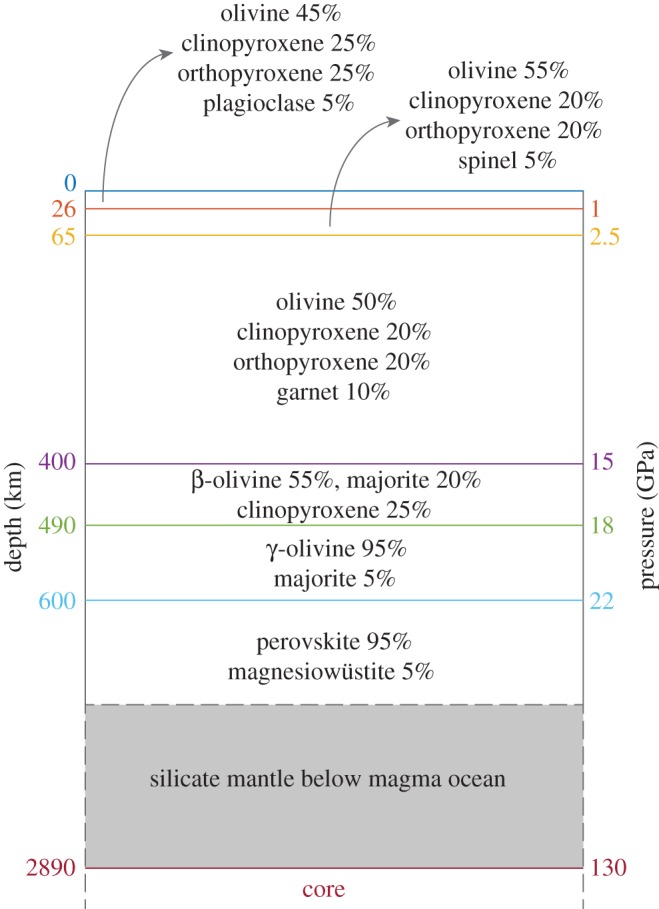
Mineral assemblages and relative abundances assumed to solidify from a terrestrial magma ocean. Trace elements and volatiles are not shown but would be incorporated into each phase in varying quantities. Adapted from figure 1 in Elkins-Tanton [20].

The mineral phase assemblages used in our code are controlled by pressure and are determined *a priori* for all models (figure 1). We assume fractional crystallization from the bottom up, accounting for the continuously evolving composition of the residual magma ocean liquid. Though a whole-mantle terrestrial magma ocean may well begin solidification at a septum above the core–mantle boundary [21], this physical detail will not substantially change the broad conclusions of these simpler models, assuming the mantle solidifies through fractional crystallization. In cases where a magma ocean solidifies from its interior in both upward and downward directions, essentially creating two magma oceans separated by a growing cumulate septum, the volume of the basal unit (located near the core–mantle boundary) would be far smaller than the overlying unit due to the cubic scaling of volume in a spherical body. Therefore, modelling a whole-mantle magma ocean with a basal magma unit that does not participate in overturn is approximately equivalent to modelling a single magma ocean with depth reaching the top of the basal magma ocean. As such, we do not model basal magma oceans in this work.

We model scenarios in which magma ocean cumulates retain either 0% or 1% interstitial liquids, depending on the model. Inclusion of interstitial liquids will increase the water content in the cumulate mantle. All water in excess of liquid saturation limits is outgassed into an atmosphere. Because the timescale of magma ocean solidification (approx. 5 Myr for a whole-mantle magma ocean and approx. 10 kyr for a shallow 500 km magma ocean [20]) is significantly shorter than thermal diffusion through the mantle, the solidified cumulates maintain their solidus temperature throughout the process. The solidus used in this study was constructed by polynomial fit to minima of the solidi from Elkins-Tanton [20] (which was determined by fitting experimental data on continental peridotite composition KLB-1 from Takahashi [44], Herzberg & Zhang [45] and Tronnes & Frost [46]) and Abe [18]. The equation describing the polynomial fit is
3.1
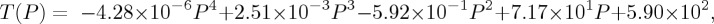

where *T* is temperature in Celsius and *P* is pressure in GPa.

Using predetermined experimental distribution coefficients, mineral-melt exchange and thermochemical parameters (see Elkins-Tanton [20] for details), we calculate mineral compositions in equilibrium with the magma ocean composition. Mineral densities are calculated based on temperature, pressure and composition. The mineral phase assemblages we use in our model are inappropriate for addressing the final few, near-surface stages of solidification because the highly evolved, late-stage magma ocean liquids would no longer solidify into an upper mantle assemblage. Therefore, in our simulations we halt magma ocean solidification at 99% completion by volume, leaving the remaining 1% unfractionated. In half of our models, these surficial cumulates do not participate in magma ocean overturn. The remaining half of our models consider the implications of their water content on the upper mantle if they do participate in overturn as an end-member scenario. Although experiments to accurately determine the composition of primordial near-surface cumulates do not currently exist, the code maintains a record of their volatile content.

### (b) Organization of water from magma ocean solidification and overturn

**Figure 2.**
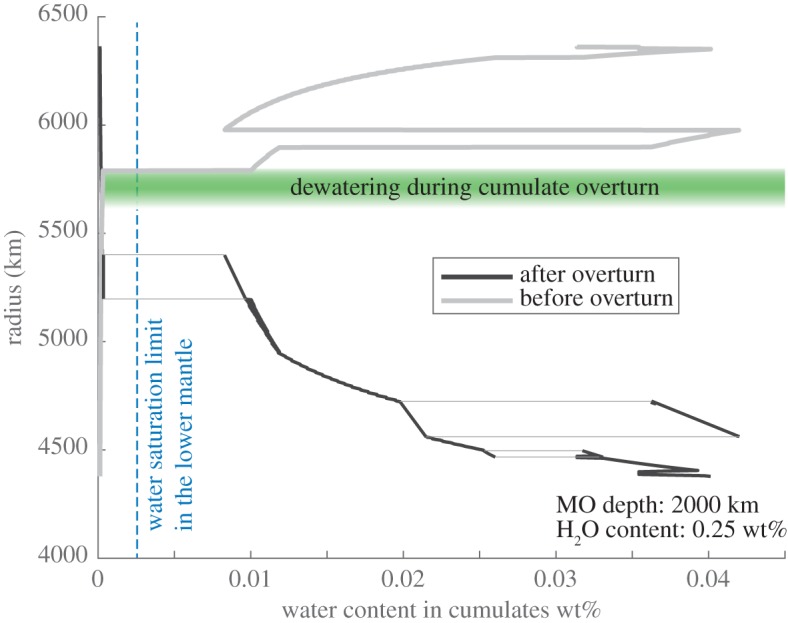
Water content of mantle cumulates from equilibrium partitioning between magma ocean liquids and fractionating solids. Interstitial liquids are not included in the model. Model results are shown for a 2000 km deep terrestrial magma ocean with an initial water content of 0.25 wt%. Light grey lines denote cumulate water content before overturn. Black lines depict the pre-overturn water contents of modelled cumulate layers, displayed at the post-overturn depth of the modelled layers. Driven by their high density, these water-rich layers would have dewatered as they sank through the transition zone. Radius ranges with two post-overturn values are regions where cumulates from two initial depths (and thus different compositions and mineralogies) have the same densities, and would settle adjacent to each other at the same radius range on some wavelength. (Online version in colour.)

Nominally anhydrous minerals in the Earth's mantle are capable of carrying petrologically significant amounts of OH within crystallographic defects (table 1 and references therein). Assuming that accretionary water exists in the Earth interior during magma ocean solidification, water will be partitioned up to saturation in solid cumulates while the excess is exsolved and degassed into a growing atmosphere [20]. Late-crystallizing magma ocean cumulates are rich in dense iron and incompatible elements. These cumulates are also enriched in water because early crystallizing lower mantle minerals (dominantly perovskite and post-perovskite) have water saturation values a factor of approximately 100 times lower than upper mantle minerals such as olivine and pyroxene (figure 2). In particular, α-, β- and γ-olivines have water saturation levels ranging approximately between 1 wt% and 3 wt% at pressure–temperature conditions approximating the upper mantle. By contrast, the lower mantle minerals perovskite and post-perovskite have water saturation levels approximately less than 0.01 wt%. Furthermore, perovskite and post-perovskite have water solid-melt partition coefficients approximately 10–100 times lower than olivine and pyroxene (table 1), further limiting the extent to which these minerals can retain water. Note that these values may also depend on ambient pressures, temperatures and variations in the Fourier transform infrared spectroscopy procedures used by different research groups to determine water content (see Keppler & Bolfan-Casanova [59] for review).

**Table 1.** Solid-melt distribution coefficients and water saturation levels for mantle minerals.

mineral	solid-melt distribution coefficient	source	H_2_O saturation (wt%)	source
α-olivine	0.002	Aubaud *et al*. [47]Grant *et al*. [48]Koga *et al*. [49]Kohn & Grant [50]	0.12	Bell *et al*. [51]Hauri *et al*. [52]Koga *et al*. [49]Kohlstedt *et al*. [53]Inoue *et al*. [54]
clinopyroxene	0.02	Aubaud *et al*. [47]Koga *et al*. [49]	0.08	Bell & Rossman [55]Bolfan-Casanova & Keppler [56]Forneris & Holloway [57]Hauri *et al*. [52]
orthopyroxene	0.02	Aubaud *et al*. [47]Koga *et al*. [49]	0.15	Bell & Rossman [55]Hauri *et al*. [52]Rauch & Keppler [58]Keppler & Bolfan-Casanova [59]
plagioclase	0.001	—	0.051	Johnson & Rossman [60]
spinel	0.02	—	0.2	—
garnet	0.0008	Bell *et al*. [51]	0.07	Bolfan-Casanova & Keppler [56]
β-olivine (Wadsleyite)	0.1	Demouchy *et al*. [61]Kawamoto *et al*. [62]	2.4	Kawamoto *et al*. [62]Kohlstedt *et al*. [53]Inoue *et al*. [54]
majorite	0.003	Bolfan-Casanova & Keppler [56]	0.0675	Bolfan-Casanova & Keppler [56]
γ-olivine (Ringwoodite)	0.03	Bolfan-Casanova & Keppler [56]Kawamoto *et al*. [62]	2.5	Bolfan-Casanova & Keppler [56]Kohlstedt *et al*. [53]Ohtani *et al*. [63]Inoue *et al*. [54]
Mg-perovskite	0.0001	Bolfan-Casanova *et al*. [64]	0.001	Bolfan-Casanova *et al*. [64]Litasov *et al*. [65]
Fe-perovskite	0.0001	Bolfan-Casanova *et al*. [64]	0.0015	Litasov *et al*. [65]
Ca-perovskite	0.0001	—	0.004	Litasov *et al*. [65]
magnesiowüstite	0.008	Bolfan-Casanova *et al*. [64]	0.0075	Bolfan-Casanova *et al*. [64]
post-perovskite	0.0001	—	0.001	—

**Figure 3.**
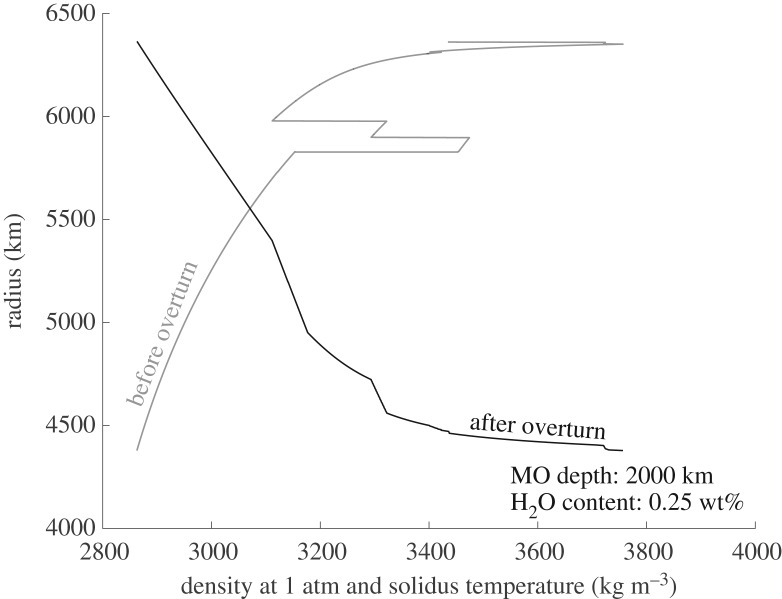
Cumulate mantle density before and after overturn for a 2000 km deep terrestrial magma ocean with an initial water content of 0.25 wt% at solidus temperatures and a reference pressure of 1 atm.

**Figure 4.**
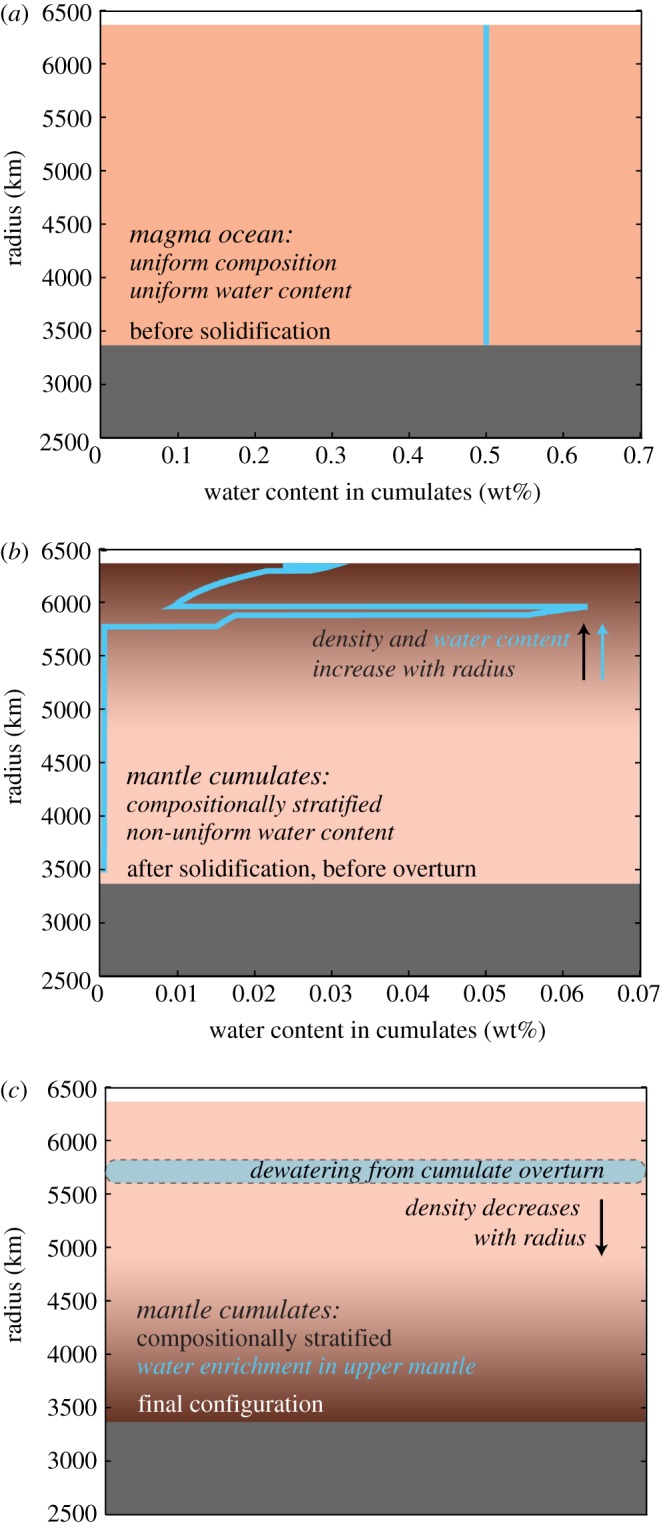
Schematic depicting the dewatering of sinking magma ocean cumulates during overturn. Panel (*a*) shows the initial state of a whole-mantle (2800 km depth) magma ocean with a uniform 0.5 wt% initial water content. Panel (*b*) shows modelled water content of cumulate layers after solidification but prior to overturn when the mantle is compositionally stratified but in an unstable density configuration. Panel (*c*) qualitatively depicts the release of water into the upper mantle as a result of hydrous cumulates sinking into the dry lower mantle during magma ocean overturn.

During overturn, dense, water-enriched upper mantle cumulates sink in the solid state to the lower mantle and lighter cumulates rise until a stable density configuration has been achieved (figure 3). As the densest upper mantle cumulates sink into the lower mantle, they enter the perovskite and magnesiowüstite stability zone. Upon crossing into the lower mantle, sinking cumulates release water in excess of the saturation limits for these minerals. We hereafter refer to this process as ‘dewatering’ (figure 4). The hydrous material immediately accumulating above the lower mantle boundary will buoyantly rise and remain in the upper mantle. The implications of the differing water capacities of the upper and lower mantle and the transition zone for modern-day thermal convection has been considered by Bercovici & Karato [66], who described the high water capacity of the transition zone and hypothesized about the dehydration that would occur in material rising into the upper mantle. Richard *et al*. [67] subsequently considered how hydrous plate slabs might dewater as they sink into the lower mantle. Here, we focus on the dehydration that would occur when freshly solidified post magma ocean material sinks downward into the lower mantle and quantify the amount of water released into the upper mantle as a result of this dewatering process. We then discuss implications for this released water in the upper mantle with regard to the onsets of mantle convection and place tectonics. Overturn timescales are viscosity dependent, but are estimated to reach 98% completion in approximately less than 10 Myr for mantle viscosities of approximately 10^18^ Pa s [68], well before the predicted onset of mantle convection approximately 25–100 Myr after magma ocean solidification and overturn [22].

### (c) Dewatering calculations

In our model, the terrestrial magma ocean (independent of depth) is divided into 100 concentric layers of equal volume. Following the solidification process, each cumulate layer has a unique composition and water content that is tracked by the code (figure 4). For magma oceans with depths greater than 600 km, we define the post-overturn lower mantle boundary as the cumulate model layer located at the depth where the corresponding pressure is 22 GPa (the boundary of the perovskite–magnesiowüstite stability zone). Cumulate layers with densities greater than that of this boundary layer will sink while those with densities less than that of the boundary layer will buoyantly rise in the upper mantle. For magma oceans with depths less than 600 km, this boundary layer is not captured in the model itself because the dense magma ocean cumulates can only sink to the bottom of the modelled magma ocean and not all the way into the lower mantle as they would in principle. For these cases, we assign layers with (atmospheric pressure-adjusted) densities *ρ* > 3100 kg m^−3^ to the lower mantle. We choose this threshold density because it represents the density corresponding to the post-overturn layer at pressure 22 GPa for a whole-mantle magma ocean. We calculate the total amount of water present in each model layer by allowing water in the magma ocean liquids to partition into the mineral in equilibrium using the partition coefficients in table 1. We then use a lower mantle water saturation limit of 2.58 × 10^−3^ wt% to determine the maximum amount of water that can be retained by that layer as it enters the perovskite-magnesiowüstite stability zone. We obtain this water saturation limit by assuming a lower mantle mineral assemblage of 64% Mg-perovskite, 8% Fe-perovskite, 8% Ca-perovskite and 20% magnesiowüstite with corresponding water saturation limits of 6.4 × 10^−4^ wt% for Mg-perovskite [64,65], 1.2 × 10^−4^ wt% for Fe-perovskite [65], 3.2 × 10^−4^ wt% for Ca-perovskite [65] and 1.5 × 10^−3^ wt% for magnesiowüstite [64]. We define the amount of dewatering as the difference between the amount of water trapped in cumulates sinking into the lower mantle and the amount of water that the lower mantle can contain up to saturation.

Because the top 1% by volume of the magma ocean remains unfractionated in our model, we cannot precisely determine the fate of those cumulates. This uppermost layer of the magma ocean, which forms during late-stage fractional crystallization, should be enriched in heavy elements and water. Several contrasting outcomes have been discussed for the evolution of this top layer (see Scheinberg *et al*. [68] and Breuer & Spohn [69] regarding possible outcomes for Mars). This layer may not participate in overturn because its high water content may enable buoyancy. It may also be cold enough to become too viscous to flow, yielding a stagnant lid regime. Alternatively, the high densities produced by heavy element enrichment may have facilitated complete or partial overturn of this layer. Because the involvement of this layer in mantle overturn is uncertain, we consider two end-member scenarios for our dewatering calculations. In the first scenario, we assume that this top (1% by volume in our models) layer will sink into the lower mantle and that its entire pre-solidification water content will undergo dewatering during overturn. This provides an upper limit on the amount of dewatering that can occur. In the second scenario, we assume that this layer does not participate in overturn at all and its water content is entirely excluded from our dewatering calculations.

## 4. Results and discussion

### (a) Amount of dewatering

**Figure 5.**
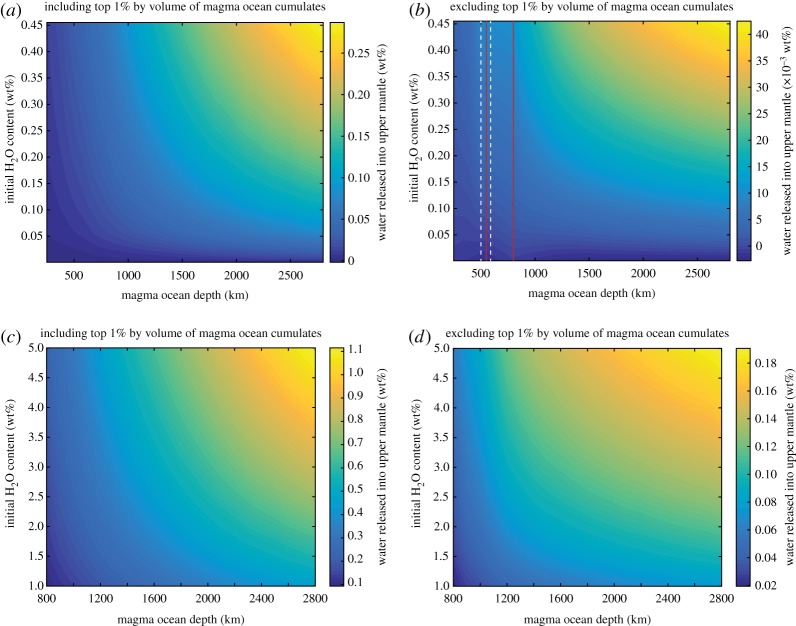
Amount of water released into the upper mantle as a result of the dewatering process for magma oceans ranging in depth between 250 and 2800 km, excluding contributions from interstitial liquids. (*a*) Calculated water release including the layer corresponding to the top 1% by volume of magma ocean cumulates for initial water contents ranging between 0.001 wt% and 0.45 wt%. (*b*) Water release excluding the top layer for magma ocean initial water contents ranging between 0.001 wt% and 0.45 wt%. Dashed white lines at magma ocean depths of 485 km and 590 km denote boundary depths at which we employed different versions of the code for different depth intervals to exclude mineral phases that do not factor in such shallow magma oceans. Because a different upper mantle mass was used to calculate the amount of dewatering for shallow magma oceans (see text) discontinuities may be observed in the amount of water released between our results for magma ocean depths between approximately 800 km and approximately 550 km (denoted by vertical red lines). (*c*) Water release including the layer corresponding to the top 1% by volume of magma ocean cumulates for initial water contents ranging between 1 wt% and 5 wt%. (*d*) Water release excluding the top layer for magma ocean initial water contents ranging between 1 wt% and 5 wt%.

**Figure 6.**
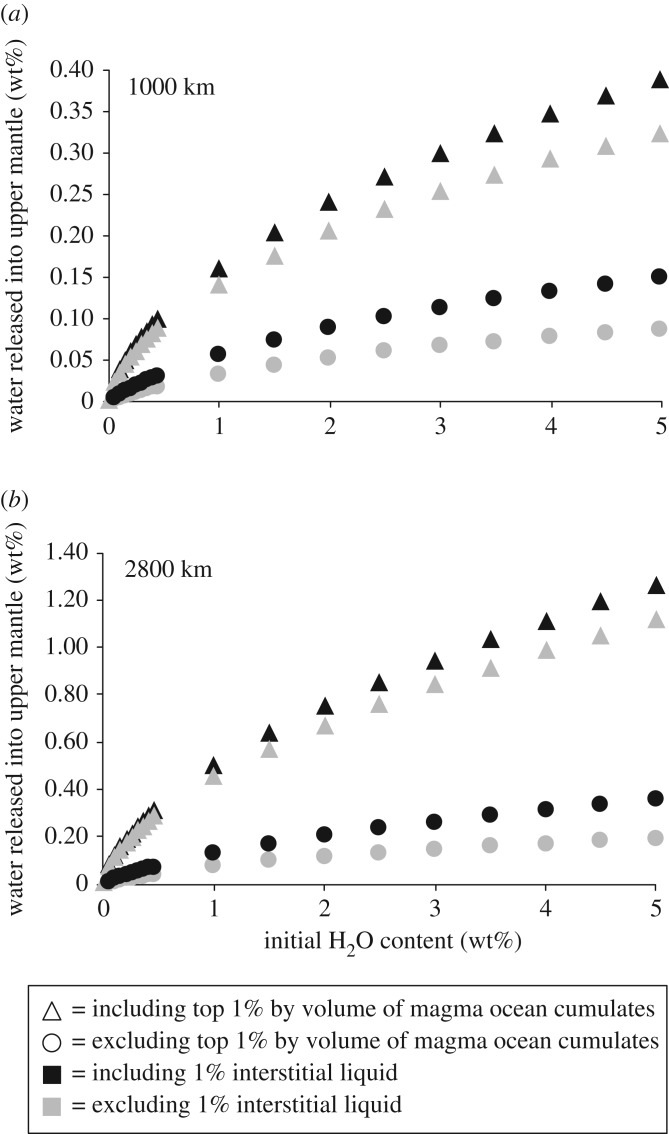
Amount of water released into the upper mantle for model runs including and excluding 1% interstitial liquids. (*a*) Models for a 1000 km deep magma ocean. (*b*) Models for a 2800 km deep magma ocean.

Initial magma ocean water contents less than 0.05 wt% did not result in dewatering of sinking cumulates. For the end-member scenario that excludes the top 1% by volume of the magma ocean from our calculations, we find that for initial magma ocean water contents in excess of approximately 0.05 wt%, the dewatering process may enrich the upper mantle with significant quantities of water (e.g. we obtained a 0.2 wt% water enrichment in the cumulate mantle for a whole-mantle magma ocean with no interstitial liquids and a 5 wt% initial water content; figure 5). Inclusion of 1% interstitial liquids in the models increases the amount of dewatering by a factor of less than 2 (figure 6). Including water contained in the uppermost 1% of the magma ocean further enriches the upper mantle (e.g. we obtained an approx. 1 wt% water enrichment for a whole-mantle magma ocean with no interstitial liquids and a 0.5 wt% initial water content; figure 5). In all cases, water enrichment increases with initial magma ocean water content and magma ocean depth. Note that even if cumulates associated with this top layer do not sink into the lower mantle, the water they contain following magma ocean solidification will either remain in the crust or upper mantle or be degassed to the atmosphere.

### (b) Fate of released water

The precise fate of the water liberated as a result of magma ocean overturn depends on percolation speeds and the relationship between upper mantle temperatures and solidi. The water will be positively buoyant because of its low density. Some of this water may recombine up to saturation with upper mantle minerals, and some may survive for some percolation distance in high-pressure polymorph phases. Water released into the upper mantle by the dewatering mechanism could trigger partial melting. The resulting hydrous silicate magmas would in turn be positively buoyant; hydrous magnesian primitive mantle melts are expected to be positively buoyant in the upper mantle [70].

The presence of water in peridotite leads to solidus depression that is roughly linear with bulk water content; for example, a bulk water content of 0.1 wt% is capable of lowering melting temperatures in the mantle by approximately 200°C for pressures ranging from 1 to 8 GPa [71]. The presence of water would also lower viscosities in the upper mantle. Hirth & Kohlstedt [72] determined that the viscosity of the MORB source region (which has a water content of 0.01 ± 0.005 wt%) is approximately 500 times lower than the viscosity of dry olivine aggregates.

Since the dewatering process described in this paper is, in principle, capable of enriching the upper mantle with potentially up to 0.1–1 wt% water (depending on the initial magma ocean water content and the fate of the top layer from our models), viscosities in the upper mantle could have been lowered to well below that of the modern MORB source region particularly for deep magma oceans exceeding approximately 1000 km in depth. The combination of lowered melting temperatures and viscosities in the upper mantle as a result of the dewatering process may have encouraged mantle convection. Rapid thermal convection in the upper mantle would increase the outward heat flux. This, in turn, can control thickness of plates, possibly inhibiting the formation of a one-plate lithosphere and setting the stage for subduction and plate tectonics [66].

### (c) Timing the onset of plate tectonics on Earth

When exactly plate tectonics began on the early Earth is debated. Harrison [73] suggests, from the chemistry of ancient zircons indicating liquid water and granite formation, that plate tectonics was operating by 4.4 Ga. Xe isotopes and magmatic compositions may indicate that plate tectonics on Earth began around the Hadean–Archean transition, around 4 Ga [74]. Geochemical similarities between variably dated 4.4–3.8 Ga mafic intrusions and rocks from a modern forearc also support subduction during the Hadean or Eoarchean eons. In contrast, inclusions in mantle diamonds may support the onset of plate tectonics as late as 3 Ga [75]. By linking the initiation of subduction to the first appearance of ophiolites, Stern [76] suggests that plate tectonics may not have commenced until as late as the Neoproterozoic.

On the modern Earth, the time necessary for a convection cell to fully complete one cycle around 500 Myr, with one ‘transit’ across the mantle taking around 100 Myr [77]. Our models suggest a rapid timescale for magma ocean solidification (upper limit of around 5 Myr for a whole-mantle magma ocean). Because primitive mantle water contents produced by our dewatering process are sometimes higher than that of modern MORB, our results are consistent with low mantle viscosities and therefore vigorous mantle convection commencing early in the Earth's history. This convection would aid the onset of plate tectonics by producing volcanism and basal stresses on the conductive lid, and by slowing the thickening of that lid. In summary, our model results support an early (Hadean or Eoarchean) onset for plate tectonic activity on the Earth, or at least ductile drip-style lithospheric recycling, such as hypothesized by Foley *et al*. [78].

### (d) Implications for the onset of plate tectonics on terrestrial planets

Ground and space-based surveys have thus far identified over 1000 planets outside our solar system. Extrasolar planets with masses up to 10 Earth masses (*M*_E_) are termed ‘super-Earths’. While the sizes and constitution of these bodies are not generally known, it is likely that several of the detected super-Earths are indeed rocky terrestrial planets with an internal structure similar to that of the Earth. The surface conditions, thermal evolution and, in turn, the habitability of these worlds is largely dependent on their tectonic regime [79].

Both observations in our solar system and fluid dynamic theory predict two regimes of planetary lid behaviour with respect to mantle convection. The first, exemplified in our solar system by the Moon, Mars, Mercury and Venus, is stagnant lid convection, when cooling through the planet's surface drives up temperature-dependent viscosity and forms a thick mechanical lithosphere that does not break or move with underlying mantle convection [80]. The second is plate tectonics, in which the lid is broken into plates that move with respect to each other, and sometimes sink back into the mantle. The transition to plate tectonics is controlled by the viscosity ratio between the lithosphere and convecting mantle [81]. Thus, the steady thickening and stiffening of the lithosphere through cooling early in the planet's evolution must be halted before the lithosphere becomes too stiff, and at the same time, mantle viscosity has to be low enough to encourage convection.

Whether or not super-Earths should be expected to exhibit plate tectonic activity remains unresolved. Analytical models suggest that plate tectonics may be inevitable on greater than 1 *M*_E_ terrestrial planets because larger planets have greater convective stresses, which enable lithospheric failure and departure from the regime of stagnant lid convection [82]. On the other hand, plate tectonics may be less likely to occur on such planets because their higher gravities would result in higher yield stresses [83]. Numerous studies have found that water is likely a stronger influence on the development of plate tectonics than planetary mass [84–86]. On the Earth, water plays a necessary role in shaping plate tectonic activity, as hydration lowers the strength of the asthenosphere, causing it to deform and flow [87]. Water also greatly influences movement of plate boundaries at depth, where sliding behaviour is attributed to the development of phyllosilicates [88]. It has been proposed that our solar system is unusually enriched in ^26^Al, causing terrestrial bodies in our solar system to be drier than most terrestrial extrasolar planets (see discussion in [89]). Because this isotope is mainly produced by supernovae and only persists for several million years before it is largely decayed, for ^26^Al to contribute significantly to planetary heating and drying a supernova must occur within the same molecular cloud as that of a newly forming solar system (i.e. within a few million years before planetesimal formation) [90]. Given these circumstances, it is possible that many extrasolar terrestrial planets accreted wetter than those within our solar system and therefore may be more likely to have plate tectonics if these bodies generated enough internal heating to differentiate and produce a silicate mantle.

With the ongoing discoveries of super-Earth extrasolar planets, the question arises whether any of these bodies could be habitable. If extrasolar planet water contents are very high an enormous steam atmosphere might be degassed from the mantle, producing waterworlds. Super-Earths would likely operate in either a stagnant lid (one-plate) or plate tectonic (multi-plate) regime. In the stagnant lid regime (i.e. absent of the efficient heat transfer capabilities of plate tectonics), heat build-up in the mantle may lead to increased melting and potentially extreme volcanism as seen on Io [91]. Plate tectonics also plays an important role in regulating planetary atmospheres. Without subduction-driven carbon sequestration, volcanic CO_2_ would build up in the atmosphere. CO_2_ molecules would then displace lighter atmospheric water higher in the atmosphere. In the upper atmosphere, water is more vulnerable to dissociation and hydrogen escape, as may have occurred on Venus [92].

As discussed above, mass-based arguments have thus far yielded conflicting results for whether or not larger (greater than or equal to 1 *M*_E_) extrasolar planets can have plate tectonics [82,83]. Our results suggest that planets with the necessary conditions to support a perovskite–magnesiowüstite stability zone (e.g. lower mantle pressures exceeding 22 GPa) may experience sufficient water enrichment in the upper mantle due to magma ocean overturn to facilitate plate tectonics. Assuming a planet with core radius equivalent to half of the total radius, a core density of 8000 kg m^−3^, and a mantle density of 3300 kg m^−3^, we estimate that the planet must have a minimum mass of approximately 0.2 *M*_E_ to yield pressures exceeding 22 GPa at the base of the mantle. Higher planetary masses are required to move the perovskite stability zone into the mid-mantle. For comparison, Mars, which lacks plate tectonics, has a mass of approximately 0.11 *M*_E_.

Therefore, our dewatering models predict that Earth-sized planets (e.g. Venus) and super-Earths would likely develop plate tectonics early. It is unknown whether Venus had plate tectonics at any stage in its past. The current absence of plate tectonics on Venus may be related to the anhydrous nature of its crust and mantle. Development of a runaway greenhouse effect on Venus would have increased surface temperatures, which then led to higher temperatures in the mantle and, in turn, partial melting and outgassing from the mantle via volcanism [93]. The short residence time of water in the venusian atmosphere (less than 300 Myr [92]) relative to the estimated 500–700 Myr intervals between hypothesized planet-scale lithospheric resurfacing events [94] suggests that the vast majority of outgassed water should have been lost due to hydrodynamic escape and therefore could not be recycled into the mantle by any known mechanism [95]. It has alternatively been proposed that Venus was dried by impact devolatilization [96] or planet-wide desiccation due to forming close to the Sun [36]. While the presence of a perovskite–magnesiowüstite stability zone may facilitate an early onset for plate tectonics, it is possible that subsequent events (such as those described above for Venus) may cause plate tectonic activity to cease.

## 5. Conclusion

Terrestrial planetary bodies are likely to acquire and retain a majority of their water throughout accretion and planet formation. Giant impacts between planetary embryos during the final phase of planet formation may have created one or more partial to whole-mantle magma oceans on the early Earth. As magma ocean solidification proceeds from the bottom-up, water is preferentially incorporated up to saturation into late-crystallizing, dense cumulates that are gravitationally unstable. This instability causes these dense cumulates to overturn and sink into the lower mantle. Because lower mantle minerals such as perovskite and magnesiowüstite have water saturation limits 10–100 times lower than upper mantle phases such as olivine and pyroxene, sinking cumulates must undergo dewatering as they enter the lower mantle. This process is potentially capable of enriching the upper mantle with up to approximately 0.1–1 wt% water. Water retained in the upper mantle as a result of the dewatering process may induce partial melting, create a damp asthenosphere, and eventually facilitate onset of mantle convection and plate tectonics on greater than 1 *M*_E_ Earth-like planets and super-Earths.
